# Structural brain change in Attention Deficit Hyperactivity Disorder identified by meta-analysis

**DOI:** 10.1186/1471-244X-8-51

**Published:** 2008-06-30

**Authors:** Ian Ellison-Wright, Zoë Ellison-Wright, Ed Bullmore

**Affiliations:** 1 Avon and Wiltshire Mental Health Partnership NHS Trust, Salisbury, UK; 2 Child and Adolescent Mental Health Service, Dorset County Hospital NHS Foundation Trust, Dorchester, UK; 3 Brain Mapping Unit, University of Cambridge, Cambridge, UK

## Abstract

**Background:**

The authors sought to map gray matter changes in Attention Deficit Hyperactivity Disorder (ADHD) using a novel technique incorporating neuro-imaging and genetic meta-analysis methods.

**Methods:**

A systematic search was conducted for voxel-based structural magnetic resonance imaging studies of patients with ADHD (or with related disorders) in relation to comparison groups. The authors carried out meta-analyses of the co-ordinates of gray matter differences. For the meta-analyses they hybridised the standard method of Activation Likelihood Estimation (ALE) with the rank approach used in Genome Scan Meta-Analysis (GSMA). This system detects three-dimensional conjunctions of co-ordinates from multiple studies and permits the weighting of studies in relation to sample size.

**Results:**

For gray matter decreases, there were 7 studies including a total of 114 patients with ADHD (or related disorders) and 143 comparison subjects. Meta-analysis of these studies identified a significant regional gray matter reduction in ADHD in the right putamen/globus pallidus region. Four studies reported gray matter increases in ADHD but no regional increase was identified by meta-analysis.

**Conclusion:**

In ADHD there is gray matter reduction in the right putamen/globus pallidus region. This may be an anatomical marker for dysfunction in frontostriatal circuits mediating cognitive control. Right putamen lesions have been specifically associated with ADHD symptoms after closed head injuries in children.

## Background

The core symptoms of Attention Deficit Hyperactivity Disorder (ADHD) are inattention, impulsivity and hyperactivity [1]. The disorder has an onset before age 7 years and affects approximately 5% of children worldwide [2].

According to one model of the neurobiology of ADHD, the aetiology involves a major genetic component. Gene variants, controlling proteins in catecholamine pathways, cause aberrant neurodevelopment of frontal brain networks supporting executive processes such as attention, memory and behavioural inhibition [3], in particular the frontostriatal circuits mediating 'cognitive control'. A common feature of the medications for treatment of ADHD is dopaminergic or noradrenergic action modulating these circuits [4], re-establishing 'cognitive control'. Low striatal dopamine levels contribute to impairments in reinforcement learning and working memory, while cortical noradrenergic deficits may contribute to other cognitive deficits [4]. In support of this model, a meta-analysis of functional imaging studies of ADHD (fMRI and PET) found that the most consistent abnormalities in patients included deficits in neural activity in frontostriatal circuits [3].

If ADHD involves abnormal neurodevelopment of brain circuits then this may be associated with structural brain changes in nodes within these circuits. There is evidence that genes controlling dopamine pathway proteins are associated with regional brain volume changes in the frontal lobe and caudate nucleus [5].

There have been many studies investigating structural brain changes in ADHD. A meta-analysis of region of interest studies of ADHD found that regional volume reductions were present in the right caudate and frontal regions [6]. However, establishing these changes as consistent associations of ADHD has remained elusive.

One problem with region of interest studies is that they usually only measure a small number of selected brain regions because the measurement process is labour-intensive. Furthermore, because of the difficulties in defining structurally complex regions of cortex, region of interest studies focus on structures that are easily definable, such as the hippocampus, at the expense of neocortical morphology. This may lead to a distorted picture of the pattern of brain changes in ADHD, with an undue concentration on relatively few cerebral structures, at the expense of a more comprehensive assessment of brain structure.

The measurement problems associated with region of interest analyses have led to the development of automated 'whole-brain' techniques for analysing structural MR images. These methods have been called 'voxel-based morphometry' because scans are analysed for structural change at the level of 'voxels' – the individual elements within a three-dimensional digital image [7]. However, the results of voxel-based morphometry studies are not always consistent. This may result from different analysis methods (including spatial transformation of the images or statistical thresholds) or variation in the samples of patients and controls (e.g. age or medication status).

In this study we conduct a meta-analysis of voxel-based morphometry imaging studies of ADHD in order to localise regions where there are structural gray matter changes and avoiding the biases of region selection which may be present in region of interest studies. On the basis of the previous meta-analyses of region of interest studies [6] and functional imaging studies of ADHD [3], we hypothesised that in ADHD there would be gray matter deficits in the right caudate and right and left frontal lobes. In the future, hypotheses regarding structural changes in ADHD may be informed by whole-brain studies including voxel-based morphometry, functional imaging and neuropsychological theories.

## Methods

### Study Ascertainment

Studies were considered for inclusion if they were published before February 2008 as an article (rather than a letter or an abstract), if they compared a group of subjects with ADHD (or Hyperkinetic disorder or subjects with high ADHD symptom scores) and a comparison group (either related or unrelated to the subjects), if they utilized voxel-based morphometry analysis of MRI datasets to investigate differences in whole-brain structure (gray matter density), and if they reported the three-dimensional co-ordinates of brain changes in stereotactic space.

Study data were excluded if insufficient data were reported to extract the number of subjects in each group, if there were fewer than six subjects in either the ADHD group or the comparison group, or if the data contributed to another publication, in which case the publication with the largest group size was selected.

A systematic search strategy was used to identify relevant studies. First, we carried out a MEDLINE search using the following keywords: ADHD, magnetic resonance imaging, voxel, SPM (statistical parametric mapping), Talairach; the search was conducted in March 2008, and no time span was specified for date of publication. Second, a manual search was also conducted of the titles of published papers in four psychiatric journals for the period February 2007 to February 2008: *Journal of Child Psychology and Psychiatry*, the *American Journal of Psychiatry*, *Archives of General Psychiatry *and *Biological Psychiatry*. Finally, we searched the reference lists of the studies identified for inclusion. Table 1 lists the articles included in the meta-analysis.

**Table 1:** Studies included in the Analyses.

**Study [Reference]**	**Patient group diagnosis**	**Male/Female/Both**	**Number of patients**	**Number of controls**	**Mean age of patients (years)**	**Patients receiving medication**
Brieber et al [15]	ADHD (DSM-IV)	M	15	15	13.1	10 prescribed methylphenidate
Carmona et al [16]	ADHD (DSM-IV TR)	B	25	25	10.8	All prescribed methylphenidate
McAlonan et al [17]	ADHD (DSM-IV)	M	28	31	9.9	All prescribed methylphenidate
Overmeyer et al [18]	ADHD (DSM-IV)	B	18	16	10.4	16 prescribed methylphenidate, 1 desipramine, 1 D-amphetamine
Van't Ent et al [19]	High-low concordant twin pairs: Scored by attention problem scale of Child Behaviour Checklist (CBCL-AP)	B	6	34	15.4	Unmedicated
Van't Ent et al [19]	Discordant twin pairs: Scored by attention problem scale of Child Behaviour Checklist (CBCL-AP)	B	10	10	15.0	Unmedicated
Wang et al [20]	ADHD (DSM-IV)	M	12	12	13.4	Not stated
TOTAL			114	143		

All studies compared a patient group with a comparison group using voxel-based morphometry of gray matter in Magnetic Resonance Images (MRI).

Co-ordinates that were reported in the stereotactic space of the Montreal Neurological Institute (MNI) were converted to Talairach coordinates using the Lancaster transform (icbm2tal) in GingerALE [8]. Talairach co-ordinates that had been generated by the Brett transform applied to statistical parametric mapping MNI co-ordinates were transformed back to MNI space in GingerALE and then to Talairach space using the Lancaster transform.

### Statistical Analysis

Meta-analyses were performed using the Talairach stereotactic coordinates derived from the studies listed in Table 1. For the primary analysis, each study was equally weighted. Secondary analyses were performed, weighting the studies by sample size (the square root of the study participant numbers).

Meta-analyses were carried out using a C++ program which modified the technique of Activation Likelihood Estimation (ALE) [8-10] by incorporating the sum-rank method developed in Genome Scan Meta-Analysis (GSMA). Genome Scan Meta-Analysis was developed to deal with the diverse marker systems used in genome scan studies [11,12] and permits weighting of studies in the meta-analysis, e.g. based on sample size. This modification of ALE treats the spatial conjunction of co-ordinates from separate studies as more significant than conjunction of co-ordinates from a single study. The probability values can then be interpreted on an image-wide basis after correction for multiple testing [13]. We used the False Discovery Rate for this step, a method which controls the proportion of type 1 errors (false positives) among significant results [14].

For each study, the reported loci of maximal anatomical difference were modeled as the peaks of three-dimensional Gaussian probability density functions with full-width half-maximum of 7 mm, within a brain mask of 210 069 voxels of linear dimension 2 mm. The voxels in this probability image were then ranked from 210 069 (highest probability) to 1 (lowest probability), giving voxels of equal probability a mean rank. This created a rank image for each study which was smoothed with a 7 mm Gaussian filter. These study images were then summated to create a sum-rank image.

A null distribution for the sum-rank image result was derived by 10 000 permutations of the same process, but using an equal number of co-ordinates for each study derived from a random uniform distribution of coordinates within the brain mask. The voxel-wise probability of a sum-rank under the null hypothesis was calculated as the proportion of permutations giving a value equal or greater then the actual value. The data set being tested was included in the ranking of all known outcomes [12]. The probability maps for the sum-rank images were ordered in magnitude and thresholded, controlling the false discovery rate at P < 0.05.

## Results

**Figure 1:**
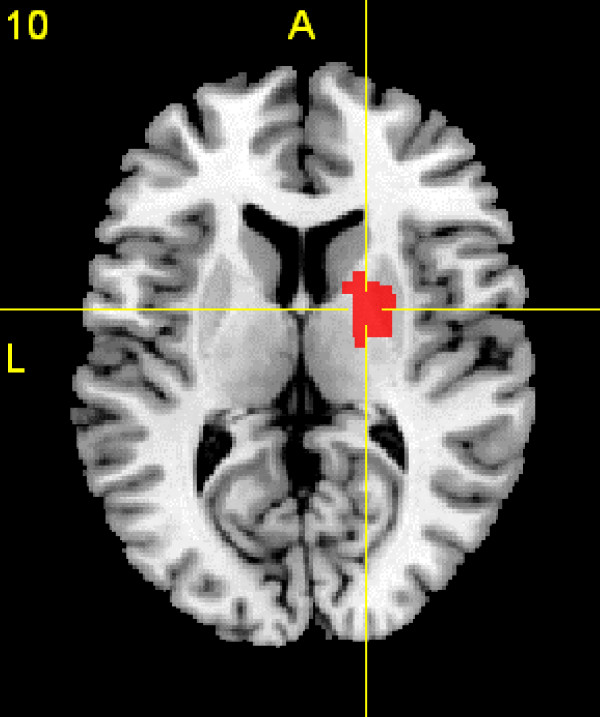
**Gray Matter Decreases in ADHD**. A transverse section at Talairach space level z = 10 showing gray matter reduction in ADHD in the right putamen/globus pallidus region, displayed on a template brain. The right side of the section represents the right side of the brain. Significant clusters were thresholded with a false discovery rate (FDR) at P < 0.05.

**Figure 2:**
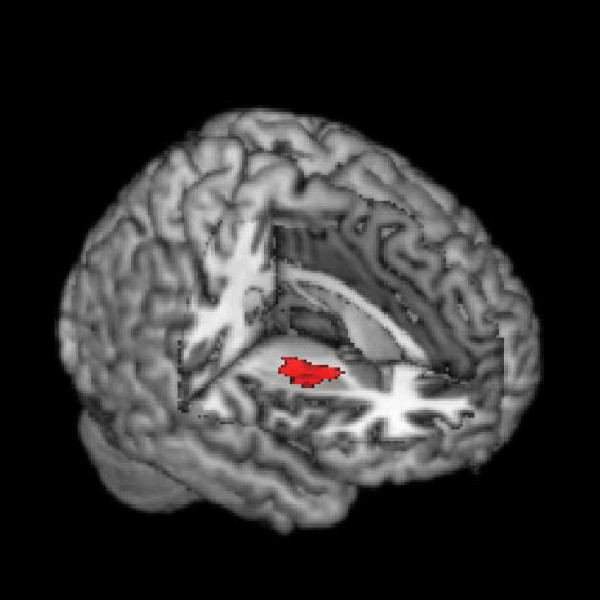
**Gray Matter Decreases in ADHD**. Gray matter signal decrease in ADHD in the right putamen/globus pallidus region, displayed on a three-dimensional rendered brain with right frontal lobe removed.

A total of 6 articles [15-20] were identified for inclusion in the meta-analysis (Table 1); the articles selected for each meta-analysis are listed in Table 2. The gray matter decreases in ADHD are displayed on brain templates (Figures 1, 2) using the Mricron software program [21].

**Table 2:** Studies available for meta-analysis.

Meta-Analysis	Gray matter signal change	Number of studies	Number of patients	Number of comparison subjects	Number of co-ordinates	Studies included [references]
A	Decrease	7	114	143	59	15, 16, 17, 18, 19, 20
B	Increase	4	43	71	17	15, 19, 20

### Gray matter decreases in ADHD

The 6 articles provided 7 studies with data on gray matter decreases in ADHD. One article [19] included a comparison of high versus low symptom score concordant monozygotic twin pairs and a comparison of high-score versus low-score discordant monozygotic twins. The results from these comparisons were included as two separate studies, as they involved separate groups of subjects.

These studies included a total of 114 patients with ADHD (or related disorders) and 143 comparison subjects and provided 59 co-ordinates for gray matter decreases.

Meta-analysis of the co-ordinates from these studies (unweighted by sample size) identified a single cluster of gray matter decrease in ADHD subjects compared with controls on sum-rank analysis. This was located in the right putamen/globus pallidus region (Talairach co-ordinates of the maximum sum-rank at x = 20, y = -2, z = 10 with voxelwise p = 0.0001; cluster size 3896 mm^3^). By comparison, analysis of the co-ordinate data using an ALE approach identified 30% fewer voxels with voxelwise p = 0.0001 and this ALE result was not significant over the whole image with the False Discovery Rate set at P < 0.05.

A similar result to the unweighted meta-analysis was obtained when the meta-analysis was repeated, weighting studies by sample size, using the square root of the study participant numbers as the weighting value (Talairach co-ordinates of the maximum sum-rank at x = 22, y = -4, z = 10 with voxelwise p = 0.0001; cluster size 4024 mm^3^).

### Gray matter increases in ADHD

There were no regions where the meta-analysis identified gray matter increases in ADHD subjects compared with controls.

## Discussion

The meta-analysis identified gray matter reduction in ADHD in the right putamen/globus pallidus region. No regions were identified with gray matter increases in ADHD.

The putamen, globus pallidus and caudate nucleus are components of the corpus striatum. The putamen and caudate nucleus are joined anteriorly and have histological similarities. The globus pallidus and putamen are separated by a thin lamina of white matter (the external medullary lamina). In functional terms, corticostriatal circuits are thought to involve projections from cortex to caudate/putamen and back to the cortex via the globus pallidus and thalamus. The activity of these circuits is modulated by dopaminergic projections to the striatum from the substantia nigra.

Evidence from a number of sources has implicated the striatum in the pathology of ADHD [22] including clinical studies of children with head injuries and strokes, structural imaging studies and functional imaging studies.

A study of 76 children who suffered closed head injury with no prior history of ADHD identified 15 who developed ADHD symptoms. The children who developed ADHD had more lesions localized in the right putamen [23]. Another study of ADHD symptoms in twenty-five children with focal stroke lesions found that the symptoms were most commonly associated with lesions of the posterior putamen [24]. Right hemisphere stroke in two children causing ADHD, obsessive compulsive disorder and Tourette symptoms also involved the basal ganglia [25].

The meta-analysis of region of interest studies of ADHD found a significant regional volume reduction in the right caudate [6]. On anatomical grounds, a reduction in right caudate volume may be associated with right putamen gray matter loss. The region of interest meta-analysis measured the caudate volume in six studies but the globus pallidus in three studies and the putamen in only two, so there was less power to detect putamen or globus pallidus changes. It found bilateral putamen and globus pallidus volume reductions in ADHD, although these differences did not reach statistical significance.

There is evidence for white matter abnormality in ADHD in the region of the right basal ganglia. An investigation of white matter structure in familial ADHD found an association between a measure of myelination (fractional anisotropy) in right frontostriatal fibre tracts and both functional activity in the frontal gyrus and caudate and performance of a go/nogo task [26]. Disruption in frontostriatal fibre tracts in ADHD may be a consequence of abnormal neurodevelopment in the basal ganglia.

Functional imaging studies have also detected abnormalities in the striatum in ADHD. In adults with ADHD, positron emission tomography studies have found lower dopamine D2/D3 receptor availability in the left caudate in ADHD [27]. Children with ADHD who responded to methylphenidate, compared to non-responders, were found to have higher cerebral blood flow on SPECT scanning in several brain regions including the right putamen [28]. Children with ADHD were also found to have reduced N-acetylaspartate/creatinine (NAA/Cr) ratios in the bilateral globus pallidus on proton magnetic resonance spectroscopy [29].

While activation patterns in functional MRI studies depend on the nature of the task and performance, a number of studies have identified hypoactivation of striatal networks in ADHD. Children with ADHD were found to show less activation in a right striatal-parietal network while performing a mental rotation task [30], less frontal-striatal activation in an executive control task [31], less activation of a frontal-striatal-parietal network during an interference suppression task [32], and less frontal-striatal activation in a response inhibition task [33].

When adolescents with ADHD performed a task involving a monetary incentive delay task, fMRI revealed reduced striatal activation during reward anticipation [34]. Conversely a fMRI study of children with a temperamental style of 'behavioural inhibition', who were shy and anxiety-prone and might be expected to have opposite problems to those with ADHD, found they showed increased striatal activation in response to monetary incentives [35].

Finally, the right putamen is part of a circuit whose dysfunction has been linked to human spatial neglect [36]. There is neuropsychological evidence that children with ADHD-type behaviours show relative left-sided neglect in visual awareness [37] which may indicate dysfunction in this brain circuit.

There are some important limitations of this meta-analysis. Firstly, only a small number of studies have examined brain structure in ADHD using voxel-based morphometry. The gray matter reduction in the right putamen/globus pallidus region was identified as significant because of the conjunction of co-ordinates from four studies [15,17,18,20] out of seven (and one study [19] which identified a region between the right insula and right putamen). Therefore, the support from these voxel-based studies should be regarded as important evidence for a structural brain change in this region but requiring replication in future samples.

A second limitation is that given the small number of studies in the meta-analysis, the power to detect changes was small. Heterogeneity among the studies (e.g. differences in subject gender, medication and diagnostic criteria) may also have reduced the power of the meta-analysis to detect differences. Therefore, although we did not confirm our original hypothesis of gray matter decreases in the right caudate and frontal lobes, there may be structural changes in ADHD which are present but were not identified. Therefore, we can not rule out the possibility that the gray matter decrease in the putamen/globus pallidus was bilateral (but only detected on the right), or that other striatal decreases were present (e.g. in the caudate), or that other brain regions were affected (e.g. the frontal lobes). However, one advantage of voxel-based studies is that all regions of the brain are analysed and so there is no *a priori *bias in region selection (which may occur with region of interest studies).

A third limitation is that the meta-analysis does not have the spatial resolution to differentiate between a gray matter reduction in the putamen and globus pallidus. Although they are both components of the lentiform nucleus [38,39] there are important differences in their neural connectivity. The striatum (caudate nucleus and putamen) sends efferent projections to the globus pallidus (which is divided into external and internal segments). It has been hypothesised that striatal cells disinihibit the thalamus (facilitating actions) via the globus pallidus interna (direct pathway) or increase inhibition of the thalamus (opposing actions) via a circuit including globus pallidus externa, subthalamic nucleus and globus pallidus interna (indirect pathway) [4]. Therefore a deficit in either putamen or globus pallidus could cause abnormalities in these cortical-striatal-pallidal-thalamic pathways, although at separate points and potentially with different consequences.

A fourth limitation is that there may be publication bias in the voxel-based morphometry literature on ADHD. Studies giving negative results may be less likely to be published than studies giving positive results (sometimes referred to as the 'file drawer problem', since negative studies are not published but remain in researchers' file drawers). Negative studies should not affect the results of this meta-analysis (since it analyses the spatial distribution of published co-ordinates) but a bias in favour of publishing studies with co-ordinates which the researchers considered to be in interesting locations could bias the meta-analysis to detect changes in these locations. Although the meta-analysis did not incorporate the significance level of the results returned in different component studies, one advantage of the Genome Scan Meta-Analysis approach is that it provides a systematic method for integrating studies with different analysis methods and statistical thresholds [12].

Finally, the gray matter reduction in the subjects with ADHD may be modulated by medication. Most of the studies in this meta-analysis included patients receiving stimulant treatment. It is possible that methylphenidate (an indirect dopamine agonist) may contribute to basal ganglia volume decreases in broadly the same way that antipsychotics (dopamine antagonists) may cause basal ganglia volume increases in schizophrenia [40,41]. There is evidence that methylphenidate causes acute changes in the T2 magnetic resonance signal in the putamen after a single dose [42].

## Conclusion

In summary, this study demonstrates the application of a novel meta-analysis technique incorporating elements of Activation Likelihood Estimation and Genome Scan Meta-Analysis. The combination of these methods has theoretical advantages over the standard Activation Likelihood Estimation system, because extra significance is given to the spatial conjunction of co-ordinates when they derive from different studies (rather than a single study) and results can be weighted by sample size.

The meta-analysis identified a right putamen/globus pallidus regional gray matter reduction in structural MRI studies of ADHD. The role of the right putamen in ADHD is supported by clinical studies of brain-injured children which have found a high incidence of right putamen lesions associated with the development of ADHD symptoms. Current neurobiological models of ADHD postulate frontostriatal dysfunction as a key component of the disorder and this may account for structural change in the right putamen/globus pallidus region during neurodevelopment.

The presence of a structural brain change in this disorder provides an impetus to further research to establish whether the change is pathological or iatrogenic and whether it may be of diagnostic utility.

## Competing interests

IEW and ZEW disclose that they have no competing interests. EB is employed half-time by GlaxoSmithKline (GSK) and half-time by the University of Cambridge and is a stockholder in GSK.

## Authors' contributions

IEW developed the meta-analysis method, wrote the software and drafted the manuscript. ZEW participated in the ascertainment of studies, extraction of data and provided clinical expertise on Attention Deficit Hyperactivity Disorder. EB participated in the design and coordination and helped to draft the manuscript. All authors read and approved the final manuscript.

## Pre-publication history

The pre-publication history for this paper can be accessed here:


